# The role of tissue oxygenation in obesity-related cardiometabolic complications

**DOI:** 10.1007/s11154-024-09910-z

**Published:** 2024-09-19

**Authors:** Geng Li, Ruth C.R Meex, Gijs H. Goossens

**Affiliations:** Department of Human Biology, Institute of Nutrition and Translational Research in Metabolism (NUTRIM), Maastricht University Medical Centre+, PO Box 616, Maastricht, 6200 MD The Netherlands

**Keywords:** Obesity, Oxygen, Hypoxia, Metabolism, Insulin resistance, Chronic diseases

## Abstract

Obesity is a complex, multifactorial, chronic disease that acts as a gateway to a range of other diseases. Evidence from recent studies suggests that changes in oxygen availability in the microenvironment of metabolic organs may exert an important role in the development of obesity-related cardiometabolic complications. In this review, we will first discuss results from observational and controlled laboratory studies that examined the relationship between reduced oxygen availability and obesity-related metabolic derangements. Next, the effects of alterations in oxygen partial pressure (pO_2_) in the adipose tissue, skeletal muscle and the liver microenvironment on physiological processes in these key metabolic organs will be addressed, and how this might relate to cardiometabolic complications. Since many obesity-related chronic diseases, including type 2 diabetes mellitus, cardiovascular diseases, chronic kidney disease, chronic obstructive pulmonary disease and obstructive sleep apnea, are characterized by changes in pO_2_ in the tissue microenvironment, a better understanding of the metabolic impact of altered tissue oxygenation can provide valuable insights into the complex interplay between environmental and biological factors involved in the pathophysiology of metabolic impairments. This may ultimately contribute to the development of novel strategies to prevent and treat obesity-related cardiometabolic diseases.

## Obesity: a gateway to other chronic diseases

Obesity is a chronic complex disease that is defined as excessive or abnormal fat storage that presents a risk to health [1]. Importantly, obesity increases the risk of several other chronic non-communicable diseases, including cardiovascular disease, type 2 diabetes mellitus (T2DM) and several types of cancer [2]. Moreover, recent evidence indicates that obesity also has a significant impact on communicable diseases, since obesity is an independent risk factor for worse clinical outcomes in patients with coronavirus disease 2019 (COVID-19) [3–5]. Globally, obesity has nearly tripled since 1975, with more than 40% of the adult population living with overweight (> 1.9 billion) and about 13% obesity (> 650 million) [1]. Over 2.8 million adults die each year as a result of overweight/obesity [1], and its economic impact is tremendous [6].

Many factors have been implicated in the etiology of obesity as well as the complications associated with this disease. Changes in the availability of oxygen in the tissue microenvironment of metabolic organs is one of the factors that may exert a key role in obesity-related cardiometabolic complications. In this review, we will first discuss findings from observational studies performed in high-altitude regions, as well as controlled laboratory studies that investigated the effects of hypoxia exposure on whole-body glycemic control. Subsequently, we will address how changes in the amount of oxygen that is present in the tissue microenvironment may affect physiological processes in key metabolic organs, including the adipose tissue (AT), skeletal muscle (SM) and liver, and how this may relate to metabolic perturbations in obesity. A better understanding of the metabolic impact of altered tissue oxygenation can provide valuable insights into the complex interplay between environmental and biological factors involved in the pathophysiology of obesity-related metabolic derangements and may contribute to the development of novel strategies to prevent and treat obesity-related cardiometabolic complications.

## The effects of high-altitude exposure on glucose homeostasis: a role for hypoxia?

Oxygen is essential to maintain human life [7]. Oxygen enters the circulation through the respiratory system and is subsequently transported, mainly bound to hemoglobin within red blood cells, to other organs in the body. Here, oxygen diffuses into the cells, allowing energy production through oxidative phosphorylation in the mitochondria. While an appropriate balance between oxygen supply and demand is crucial for cell survival, alterations in tissue pO_2_ have previously been linked to metabolic changes [8].

**Fig. 1:**
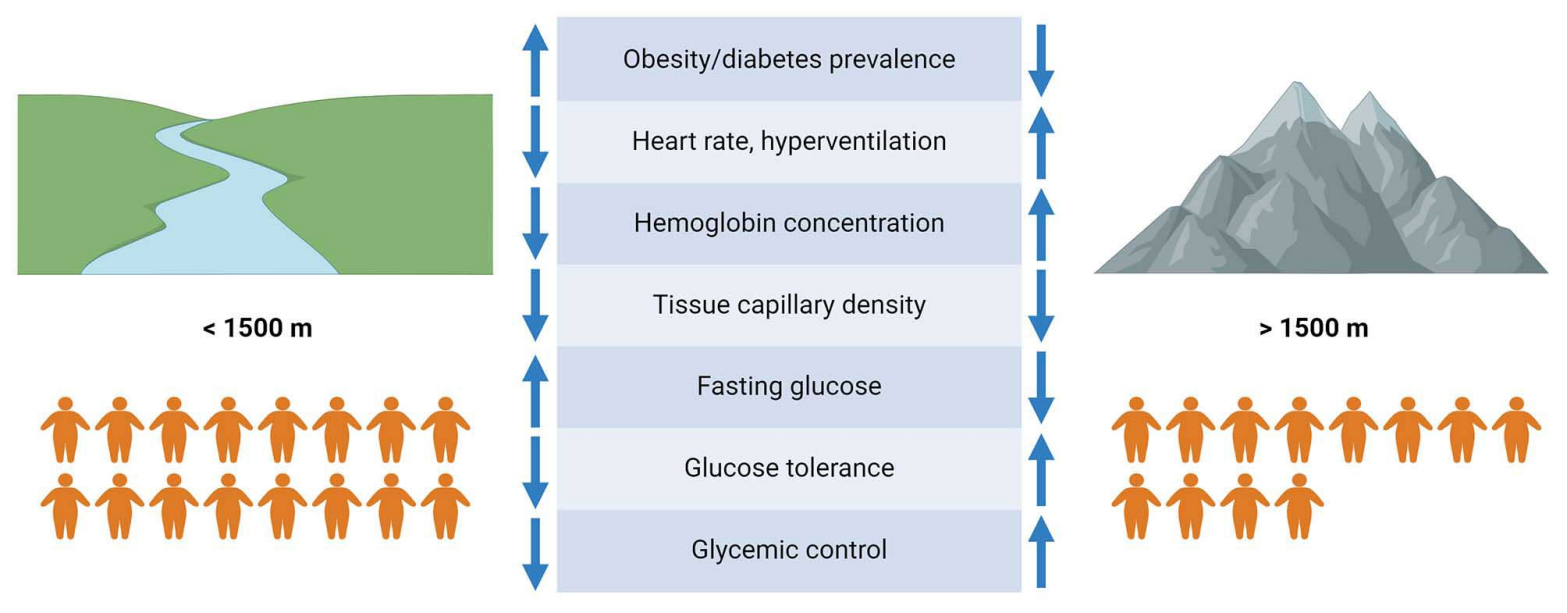
The effects of high-altitude exposure on the cardiovascular system and glucose homeostasis in humans. Figure created with BioRender.com

It has been shown that a hypoxic environment, as present in high-altitude regions due to lower atmospheric pressure, triggers a number of adaptive responses such as increased heart rate, hyperventilation, elevated hemoglobin concentration, and a higher tissue capillary density [9] (Fig. 1). Importantly, chronic hypoxia exposure also seems to have profound effects on metabolic homeostasis [10, 11]. Residents living at high altitudes have lower fasting glucose levels and better glucose tolerance compared to individuals living at sea level. In agreement with this, the prevalence of both obesity and T2DM is lower in high-altitude regions, independent of multiple risk factors (Fig. 1) [12, 13]. Hence, it is tempting to suggest that hypoxia exposure may protect against the development of obesity-related metabolic derangements and cardiometabolic diseases. Nevertheless, there are several confounding factors that may also play a role in the beneficial metabolic effects that are seen in residents living at high altitudes, including a more active lifestyle, a different eating pattern, different housing conditions, reduced urbanization, and genetic factors [10, 11, 14]. When studying the effect of exposure to very high altitudes on blood glucose levels in healthy lowlanders mixed results have been found, with studies reporting improvements [15], deteriorations [16, 17], or no notable changes [18] in glycemic control. Though, the studies that observed a worsening of glycemic control following high-altitude exposure reported that the increase in glucose levels was an acute and transient effect due to changes in hormone levels, including elevations in cortisol and catecholamines [16, 17]. Upon acclimatization, the initial increase in glycemia is often attenuated, and may even decrease to below pre-exposure levels [16, 17], suggesting that exposure to high altitude indeed has a beneficial effect on glycemic control.

The studies discussed above, however, do not allow conclusions about the importance of altered pO_2_ in the metabolic alterations and the lower prevalence of cardiometabolic diseases found at higher altitudes, due to several confounding factors. Controlled laboratory studies provide more insight into the specific contribution of altered pO_2_ to changes in glucose homeostasis, as discussed in more detail below.

## The effects of hypoxia exposure on glucose homeostasis

In recent years, several studies investigated the effects of (intermittent) hypoxia exposure on glycemia, reporting both beneficial metabolic adaptations as well as neutral metabolic effects. Acute short-term hypoxia exposure (14.6% O_2_ for 1 h) [19] as well as ten nights of hypoxia exposure (15% O_2_, 10 h/night) [20] improved insulin sensitivity in humans. Furthermore, intermittent hypoxia exposure for 1 h (5 × 6-min breathing of 13% O_2_, with each hypoxic cycle separated by 6-min recovery at normoxia) was found to improve blood glucose levels compared with normoxia exposure in individuals with T2DM [21]. Several studies have also indicated that hypoxia exposure during exercise induces beneficial effects on glycemic control [19, 22, 23]. For example, six weeks of exercise in hypoxic conditions (15% O_2_, 3 times per week for ~ 1 h) improved glucose tolerance and insulin levels in adolescents with obesity [23], as well as insulin sensitivity in men with obesity and metabolic syndrome [22].

**Fig. 2:**
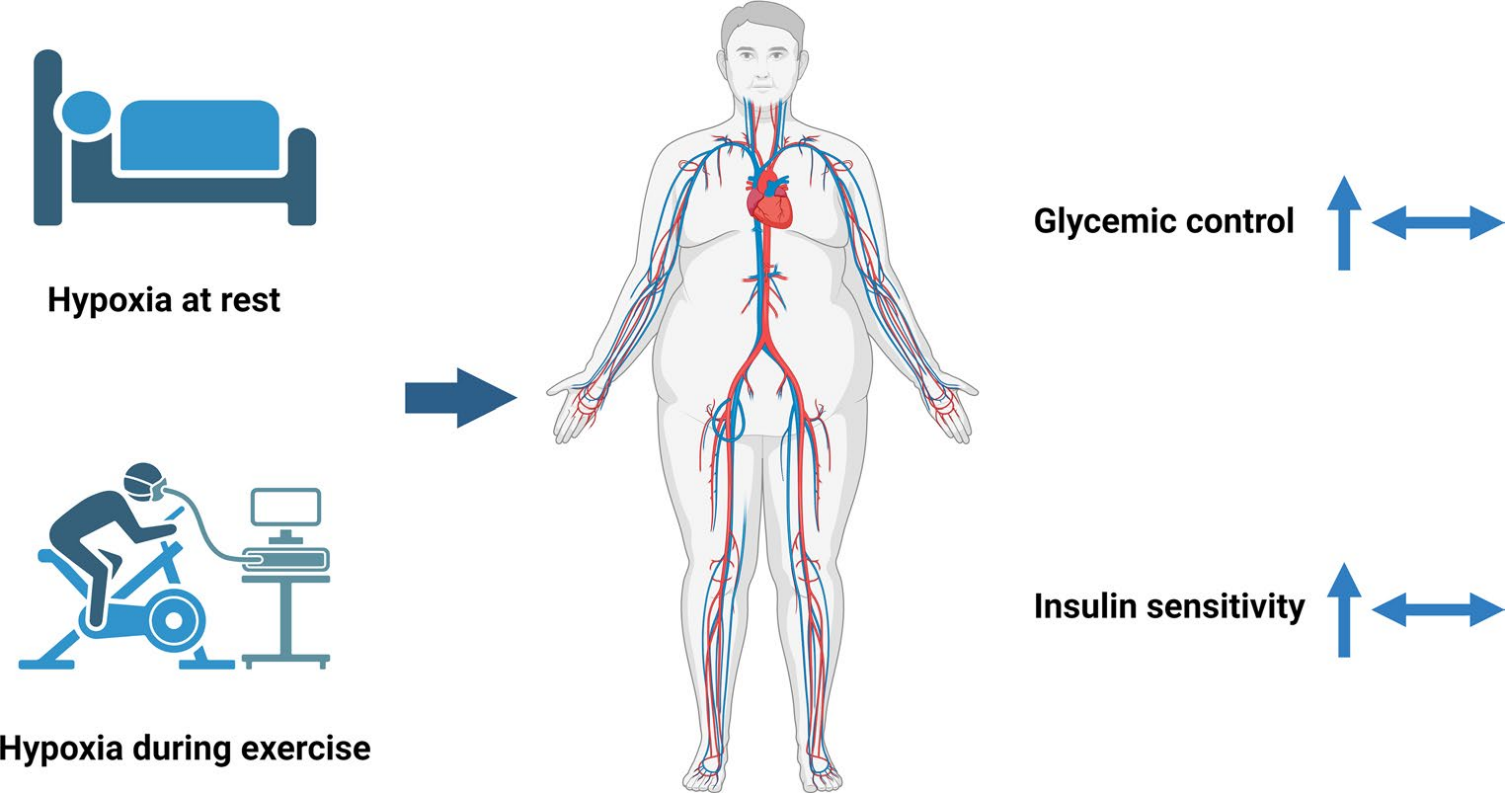
The effects of hypoxia exposure on glucose homeostasis in humans. Figure created with BioRender.com

In contrast, a number of studies found no significant effects of hypoxia exposure at rest [24–26] or after a hypoxic exercise intervention [24, 27–29] on glucose regulation and/or insulin sensitivity. The lack of effect after hypoxic exercise at the same relative training intensity compared to normoxic exercise may be due to the fact that the absolute training intensity is decreased in hypoxic conditions. Indeed, we have recently demonstrated that hypoxic exercise at similar relative exercise intensity (i.e. lower absolute workload) had no significant effects on mean 24 h glucose concentrations and glycemic variability compared to normoxic exercise in men with overweight/obesity [29]. Interestingly, however, the latter study did show that a more pronounced reduction in systemic oxygen saturation during hypoxic exercise was associated with lower 24 h and daytime glucose concentrations [29]. As explained in a recent systematic review, conflicting results of hypoxia exposure on glucose homeostasis can at least partly be explained by heterogeneity in study populations, intervention duration and/or the lack of a control group [14]. Taken together, current evidence suggests that mild hypoxia exposure may have beneficial or neutral effects on glucose homeostasis (Fig. 2). To better understand the effects of hypoxia exposure on glucose homeostasis, more detailed investigations of the effects of altered pO_2_ at the tissue and cellular level are needed, especially in key metabolic organs such as the AT, SM and liver.

## The impact of altered oxygen availability in metabolic organs

Each tissue in the body has its own range of pO_2_. Compared to pO_2_ in arterial blood (~ 104 mmHg), the average pO_2_ at the tissue level is ~ 40–50 mmHg (~ 5–6%). However, pO_2_ is much lower in the retina (2–15 mmHg; ~0.5-2%), brain (0.4 − 10mmHg; ~0-1.5%) and solid tumors (0–10 mmHg; ~0-1.5%) [30]. The physiological pO_2_ of AT in humans ranges between ~ 25–85 mmHg (~ 3–11%) [8, 31], whereas the pO_2_ level in SM ranges from ~ 8–25 mmHg (1–3%) [32–35]. Due to more variation in oxygen supply (blood flow) and consumption (metabolic rate) throughout the day, pO_2_ levels may fluctuate more in metabolically active tissues such as SM compared to other tissues [36]. Heterogeneity in pO_2_ within organs may also be present, dependent on the anatomical location. Importantly, alterations in the oxygenation of metabolic organs have been linked to changes in tissue metabolism, as discussed in detail below.

### The role of altered oxygen tension in adipose tissue

The AT serves as the primary energy storage depot and plays an important role in buffering the daily influx of excess calories. Abdominal fat mass expansion results in enlargement of adipocytes (hypertrophy) [37], leading to impaired clearance of meal-derived triglycerides and lipid spillover into the circulation in individuals with obesity [38]. The excessive flux of lipids that are directed towards other tissues results in ectopic lipid accumulation in SM, pancreas, liver and heart, thereby contributing to insulin resistance, impaired insulin secretion and related cardiometabolic impairments [37, 39, 40].

Notably, when excessive energy influx leads to expansion of the AT, inadequate angiogenesis may hamper sufficient oxygen supply. Several studies have demonstrated that angiogenesis and capillary density are reduced in the AT in individuals with obesity [41–43], which may lead to lower oxygen supply to the tissue. Indeed, we have previously demonstrated that alterations in AT blood flow induced by local micro-infusion of vasoactive agents into the AT microenvironment were accompanied by concomitant changes in AT pO_2_ [43]. Interestingly, although the link between obesity and AT hypoxia has been demonstrated in rodents [44, 45], conflicting findings have been reported in human studies, showing both higher [43, 46, 47] and lower [42, 48] AT pO_2_ in individuals with obesity compared to normal weight, despite lower AT blood flow (that is, oxygen supply) [41–43], as reviewed [31]. Conflicting findings have also been reported regarding the association between AT pO_2_ and insulin sensitivity in humans [31]. Some cross-sectional studies have shown that AT oxygenation was positively associated with insulin sensitivity in individuals with obesity [43, 46], whereas others found an inverse association between AT oxygenation and insulin sensitivity in men and women, independent of adiposity [49]. To provide a better insight into the relationship between changes in adiposity and AT pO_2_, we recently performed an intervention study in humans, showing that diet-induced weight loss decreased AT pO_2_, which was accompanied by improved insulin sensitivity in humans with overweight/obesity [47]. Discrepancies in human studies may relate to differences in the study populations and/or techniques used to measure pO_2_, as well as differences in the way insulin sensitivity was measured or defined [31, 50].

#### Effects of altered oxygen tension on adipose tissue lipid metabolism

To better elucidate the effect of hypoxia on lipid metabolism in adipocytes, several in vitro studies have been performed, in which exposure to different oxygen levels has been applied either during or after adipocyte differentiation. A study that examined the effects of severe hypoxia after differentiation found that exposure of *3T3-L1* cells to 1% O_2_ for 24 h reduced free fatty acid (FFA) uptake and increased lipolysis [45]. In line with the reduction in FFA uptake following hypoxia exposure, there was a decrease in the expression of the fatty acid transport proteins (FATP) and CD36, as well as the transcription factors peroxisome proliferator-activated receptor γ (PPARγ) and CCAAT/enhancer-binding protein-α [45]. In line with this, lipolysis was markedly increased, whereas lipogenesis was reduced when adipocytes were exposed to 1% O_2_ during differentiation [51, 52].

**Fig. 3:**
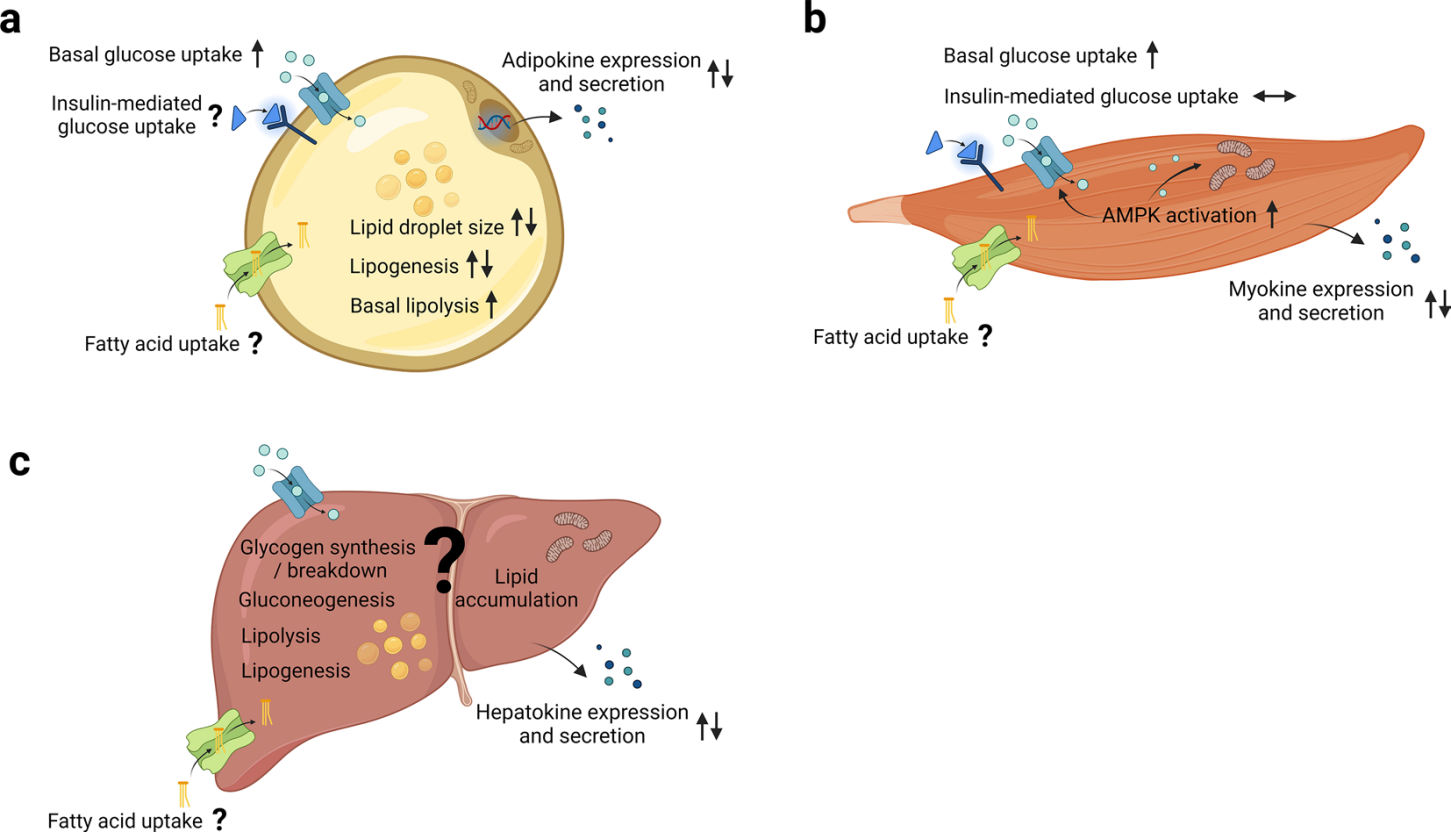
The effects of hypoxia exposure on glucose homeostasis in humans. The effects of mild physiological hypoxia exposure on metabolic and endocrine processes in (**a**) adipose tissue, (**b**) skeletal muscle and (**c**) the liver. AMPK, AMP-activated protein kinase. ↑, increased by hypoxia; ↓, decreased by hypoxia; ↔, unchanged by hypoxia; ⇅, conflicting evidence. Figure created with BioRender.com

Notably, studies investigating the effect of mild hypoxia exposure, either during or after differentiation, showed conflicting results. Whereas one study found that differentiation of *3T3-L1* adipocytes under mild hypoxia markedly increased lipogenesis and resulted in increased formation of large lipid droplets [52], another study reported that 7-days mild hypoxia exposure after differentiation resulted in a significant reduction in lipid droplet size and triglyceride content [53]. The latter study also found enhanced basal but blunted isoproterenol-induced lipolysis following hypoxia exposure [53]. Furthermore, the expression of lipogenic proteins, including fatty acid synthase, lipin-1, and PPARγ, were found to be decreased following hypoxia exposure [53]. Taken together, although conflicting results have been reported depending on the severity, duration and timing of hypoxia exposure, it is evident that altered pO_2_ has effects on both lipid breakdown and lipid synthesis in adipocytes (Fig. 3a).

#### Effects of altered oxygen tension on adipose tissue glucose metabolism

Hypoxia induces a switch from fat oxidation towards glucose utilization in adipocytes [54]. Many studies have demonstrated that acute exposure of adipocytes to hypoxic conditions leads to increased glucose uptake, glycolysis and upregulation of anaerobic enzymes to maintain intracellular energy supply, as reviewed [31]. Indeed, acute and severe exposure to 1% O_2_ has been found to increase basal glucose uptake in both human and rodent adipocytes [45, 55, 56]. However, exposure to 1% O_2_ for 24 h resulted in the development of insulin resistance in human and *3T3-L1* adipocytes [56].

study found that acute exposure to 1% O_2_ enhanced insulin-dependent and insulin-independent glucose uptake in *3T3-L1* adipocytes [57]. Interestingly, the latter study also demonstrated that repeated exposures to hypoxia (1% O_2_, 4 h/d for 4–8 days) improved insulin sensitivity, reflected by enhanced insulin-mediated glucose uptake, and increased triglyceride accumulation through enhanced protein expression of the lipogenic transcription factors PPARγ and sterol regulatory element-binding proteins(SREBP) in *3T3-L1* cells [57]. It is important to note, however, that in many of these experiments the cells were exposed to non-physiological pO_2_. We have recently demonstrated that prolonged exposure to low physiological oxygen levels (5% O_2_ for 14 days) increased basal glucose uptake in differentiated human multipotent adipose-derived stem cells [58]. In conclusion, hypoxia exposure increases basal glucose uptake, whereas effects on insulin-mediated glucose uptake in adipocytes seem dependent on the exposure conditions (that is, duration and/or severity of hypoxia) (Fig. 3a).

#### Effects of altered oxygen tension on adipokine secretion

Although AT has long been recognized as an energy storage depot, it was not until the discovery of leptin in 1994 that the concept of AT as an important endocrine organ gained acceptance [59]. Besides a role in impaired lipid metabolism, multiple lines of evidence have demonstrated that hypertrophic expansion of adipocytes is also associated with perturbations in the secretory function of AT. Increased AT expression and/or secretion of inflammatory markers, as seen in obesity, may contribute to low-grade systemic inflammation and insulin resistance [60, 61]. Of note, we have recently demonstrated that circulating immune cell populations and inflammatory gene expression in AT show distinct associations with liver and muscle insulin sensitivity [62, 63].

Several studies have shown that alterations in pO_2_ influence the endocrine function of adipocytes and/or AT. We have shown that mild intermittent hypoxia exposure in vivo in men with obesity for seven consecutive days (6 h/day) consistently reduced AT pO_2_ and altered AT protein expression, compared to normoxia exposure [26, 31, 64]. Also, multiple cell culture studies have demonstrated that the expression and secretion of adipokines are regulated by microenvironmental pO_2_. These studies, however, vary with respect to the severity of hypoxia, the total duration as well as pattern of hypoxic episodes, which seems to impact study outcomes [64]. Exposure to severe hypoxia (1% O_2_) has been shown to induce pro-inflammatory responses in adipocytes, as illustrated by the upregulation of tumor necrosis factor (TNF-α), interleukin 6 (IL-6), monocyte chemoattractant protein 1 (MCP-1), and plasminogen activator inhibitor-1 (PAI-1) gene expression, as reviewed elsewhere [30, 31]. Similarly, the expression of vascular endothelial growth factor (VEGF), which promotes angiogenesis, was stimulated by severe hypoxia in both human and rodent adipocytes [55]. Again, it is important to emphasize that many in vitro experiments have exposed cells to severe hypoxia, which may not reflect physiological conditions in humans. Application of hypoxic conditions that mimic physiological pO_2_ levels is important to better understand the metabolic and endocrine abnormalities in adipocytes in obesity. In a study investigating the impact of 24 h exposure to different pO_2_ levels in human white preadipocytes, it was observed that leptin, VEGF, and IL-6 expression increased with lower pO_2_, while adiponectin gene expression showed an opposite pattern [65]. Other studies have demonstrated that prolonged exposure to mild physiological oxygen levels decreased pro-inflammatory gene expression (i.e., IL‐6, PAI‐I, TNF-α, MCP‐1 and dipeptidyl‐peptidase‐4) and altered adipokine secretion in differentiated human adipocytes as compared to 21% and/or 10% O_2_ [58, 66]. We have previously shown that AT pO_2_ was lower in femoral compared to abdominal subcutaneous AT in postmenopausal women [58]. In addition, we found that exposing differentiated human mesenchymal stem cells from both abdominal and femoral subcutaneous AT to low physiological oxygen levels (5% compared to 21% O_2_) altered gene expression and adipokine secretion in cells from people with overweight or obesity, but not in cells derived from individuals with normal body weight [58, 67].

Further studies are warranted to examine the interaction between adipokine expression/secretion and microenvironmental pO_2_ in AT, thus providing a more comprehensive understanding of the role that altered pO_2_ may play in AT dysfunction and related cardiometabolic complications in obesity (Fig. 3a).

### The role of altered oxygen tension in skeletal muscle

SM plays an important role in whole-body glucose uptake [68]. The regulation of SM glucose disposal involves both insulin-dependent and insulin-independent mechanisms, as extensively reviewed elsewhere [14]. In individuals with obesity, however, lipid spill-over from the AT together with impairments in substrate oxidation result in the accumulation of triglycerides and toxic lipid intermediates such as ceramides and diacylglycerols (DAGs) in SM [68]. This leads to the activation of protein kinase C (PKC), which in turn blunts phosphorylation of the insulin receptor substrate, thereby impairing insulin signaling in muscle cells. As a result, insulin-stimulated translocation of GLUT4 towards the cell membrane and, consequently, glucose uptake into the myocyte will be reduced [68, 69], resulting in elevated postprandial glucose levels in. individuals with obesity if insulin secretion is insufficient [70].

#### Effects of altered oxygen tension on glucose uptake

Insulin-independent glucose uptake is partly mediated via the AMP-activated protein kinase (AMPK) pathway, which is activated under stress conditions such as exercise and hypoxia [68, 71, 72]. AMPK exerts a central role in the regulation of energy homeostasis and is involved in multiple signaling pathways, including glucose and lipid uptake, and fatty acid oxidation [72]. Once muscle cells are experiencing a low energy status, more ATP will be converted to AMP resulting in the activation of AMPK, which will result in the inhibition of ATP-consuming pathways, such as gluconeogenesis and protein synthesis, and stimulation of processes that contribute to ATP generation such as glucose uptake, glycolysis and fatty acid oxidation [73]. AMPK activation under hypoxic conditions has been demonstrated in various tissues and cell types, representing a prudent strategy to conserve energy during oxygen deprivation [74]. By reducing cellular ATP consumption, the demand for oxygen is also decreased, aligning with an adaptive response to hypoxic conditions [74].

When SM cells are exposed to hypoxia, physiological adaptation will occur to maintain tissue homeostasis (Fig. 3b). Increased glucose disposal following hypoxia exposure has been reported in animal models and primary muscle cells [75]. Available evidence suggests that hypoxia exposure as well as muscle contraction stimulates glucose uptake via insulin-independent pathways in rodent and human myotubes [14]. Indeed, we have recently found that exposure to low physiological pO_2_ levels increases insulin-independent glucose uptake, at least partly due to activation of AMPK in primary human myotubes but did not affect insulin-mediated glucose uptake [26]. In line, we have demonstrated that mild hypoxia exposure for seven consecutive days did not significantly alter adipose, hepatic and SM insulin sensitivity, assessed by the gold standard two-step hyperinsulinemic-euglycemic clamp [26].

#### Effects of altered oxygen tension on myokines

Similar to AT, SM is an important endocrine organ that produces and secretes various myokines (muscle-secreted proteins) that can influence metabolism through autocrine, paracrine, and endocrine signaling. Many myokines generated by SM seem to rely on contraction, illustrated by the fact that the intensity of physical activity influences myokine secretion [76]. Some myokines such as myostatin, leukemia inhibitory factor (LIF), IL-6 and IL-7 may play a role in conveying the protective benefits of physical exercise, particularly in conditions linked to a sedentary lifestyle [76]. Several myokines such as IL-6, IL-15, myostatin, irisin, secreted protein acidic and rich in cysteine (SPARC), and growth and differentiation factor (GDF)-15 have been shown to affect metabolism in other tissues [68, 77–80]. For example, the secretion of IL-6, SPARC and GDF-15 from muscle increases by exercise / muscle contraction and lead to activation of AT lipolysis [68, 77–80].

We recently performed a randomized cross-over study to investigate the effect of mild intermittent hypoxia exposure (15% versus 21% O_2_, 3 × 2 h/d for 7 consecutive days) on circulating myokine concentrations in individuals with obesity. Although neither acute nor 7 days of mild intermittent hypoxia exposure induced changes in plasma concentrations of several myokines in people with obesity, we found that hypoxia exposure (1% O_2_) increased the secretion of SPARC, follistatin like 1 (FSTL1) and IL-6 in primary human myotubes [81]. SPARC has been shown to improve AMPK-mediated glucose uptake [82], whereas IL-6 has been shown to regulate glucose homeostasis in SM [83]. Thus, it is tempting to postulate that decreased pO_2_ during muscle contraction may contribute to increased glucose uptake, at least partly through effects on myokines (Fig. 3b).

### The role of altered oxygen tension in the liver

The liver is another key metabolic organ that is involved in many physiological processes, including nutrient metabolism, immune function and detoxification. Changes in the delivery or utilization of oxygen may cause tissue pO_2_ alterations in different anatomical areas of the liver, which may lead to changes in metabolism. Several in vivo and ex vivo experiments in rodents support the notion that hypoxia exposure may improve metabolic functions in the liver, including regulation of glucose and lipid metabolism, as well as inflammatory responses, as outlined below.

High-altitude (~ 4300 m) chronic hypoxia ameliorated the detrimental effects of a high-fat diet (HFD) in the liver of mice, as shown by a decreased lipid accumulation and reduced expression of genes related to lipid synthesis, decreased reactive oxygen species production, upregulation of mitochondrial biogenesis and mitochondrial capacity, increased expression of carnitine palmitoyl transferase I, and increased AMPK signaling [84]. In support of these findings, chronic intermittent hypoxia exposure (10% oxygen, 1 h/d for six weeks) showed a protective effect on HFD-induced obesity in C57BL/6 mice, reflected by reduced weight gain and liver fat accumulation [85]. Moreover, in the latter study, genes related to lipolysis and thermogenesis, as well as AMPK signaling, were increased following hypoxia exposure [85]. In another study, alterations in glucose metabolism in the liver and SM were investigated in rats exposed to a hypoxic environment (10% O_2_ for 90 min), showing increased expression of genes involved in glucose utilization and gluconeogenesis [86].

Notably, most of the in vitro studies examining the effects of O_2_ levels in liver cells have been conducted under conditions of severe hypoxia rather than physiological O_2_ levels. Severe hypoxia exposure, typically exposure to 1% O_2_, has been shown to activate hypoxia-inducible factor 1-alpha (HIF-α) and subsequently stimulate the expression of PPARγ in HepG2 cells [87]. Severe hypoxia exposure (1% O_2_ for 12 h) also resulted in upregulation of HIF-2α in human L02 hepatocytes, which was accompanied by hepatic lipid accumulation [88]. In addition, intermittent hypoxia exposure (1% O_2_ for 60s alternated with 21% O_2_ for 60s during 48 h) decreased insulin-stimulated glycogen synthesis (protein kinase B (AKT) / glycogen synthase kinase-3β (GSK-3β) phosphorylation) in a time-dependent manner in liver cells, while inhibiting gluconeogenesis (forkhead box protein O1 (FOXO1) expression and phosphoenolpyruvate carboxykinase (PEPCK) transcription), independent of insulin signaling [89]. Furthermore, severe hypoxia exposure increased alpha-smooth muscle actin (α-SMA) protein expression in human hepatic stellate cells (LX-2), which serves as a marker for hepatic stellate cells activation [90]. Activated hepatic stellate cells are involved in cell proliferation, increased contractility, enhanced matrix production, and expression of a number of fibrogenic and proliferative cytokines and their cognate receptors [90, 91].

Importantly, only a limited number of studies have examined the effects of O_2_ levels that are present in the local hepatic microenvironment in (patho)physiological conditions. In the liver, oxygenated blood from the portal venules and the hepatic arterioles enters the sinusoids where oxygen is consumed, mainly by the hepatocytes, forming an oxygen gradient (“zonation”) [92]. Multiple factors, including nutrients, matrix composition, and the distribution of non-parenchymal cells are thought to contribute to this hepatocyte zonation [93]. The periportal zone (‘zone 1’) is supplied with highly oxygenated blood (pO_2_ ~ 10–12%), whereas the perivenous zone (‘zone 3’) is proximal to the central vein and receives oxygen-depleted blood (pO_2_ ~ 3–5%; 25–35mmHg) [94, 95]. Interestingly, using a human liver microphysiological system, it has recently been shown that oxidative phosphorylation was reduced and glycolysis was increased in the zone of the liver acinus with lower oxygen availability (zone 3) compared to the zone with higher oxygen availability (zone 1) [96]. In another study, exposure to 5% O_2_ has been shown to enhance the survival rate of liver sinusoidal endothelial cells (LSECs), while reducing the production of IL-6 and increasing the secretion of the anti-inflammatory factor IL-10, in comparison to exposure to ambient air [97].

While not many studies have investigated the impact of changes in pO_2_ on hepatokine secretion and liver metabolism, and the molecular mechanisms involved, effects mediated through changes in liver physiology may contribute to the effects observed following whole-body hypoxia exposure. Moreover, in pathophysiological conditions in which liver pO_2_ values might be altered such as obesity (especially in patients with obstructive sleep apnea) and metabolic dysfunction-associated steatotic liver disease (MASLD), this could underlie perturbations in metabolic/inflammatory processes in this organ. Although studies have been conducted to unravel the regulatory function of specific hepatokines, further research is necessary to better understand the involvement of pO_2_ in liver metabolism and hepatokine expression/secretion (Fig. 3c). Together, this may contribute to the identification of potential novel therapeutic strategies to prevent or treat obesity-related metabolic impairments.

## Conclusions and future perspectives

The cardiometabolic complications that often accompany obesity are the result of a complex interplay between environmental and biological factors. Intriguingly, observational studies have shown that the prevalence of obesity and T2DM is lower in high-altitude regions compared to countries close to sea level. These findings might be explained by exposure to the reduced pO_2_ in the atmosphere at high altitudes, although the contribution of other confounding factors cannot be excluded. Indeed, research of the past decades has shown that changes in pO_2_ in the metabolic tissue microenvironment seem to be involved in the development of obesity-related cardiometabolic derangements. Several laboratory studies demonstrated that hypoxia exposure has beneficial or neutral effects on glucose homeostasis. The metabolic effects of hypoxia exposure are at least partly explained by adaptations in the metabolic and/or endocrine functions of AT, SM and liver that occur as a result of alterations in microenvironmental pO_2_.

Modest changes in tissue pO_2_ may have beneficial effects on physiological processes, while other, more severe changes, have been linked to detrimental health effects. Alterations in tissue pO_2_ may occur in several disease states such as obesity, T2DM, cardiovascular diseases, chronic kidney disease, chronic obstructive pulmonary disease and obstructive sleep apnea [27, 86, 98–100]. A lower oxygen availability in the tissue (relative oxygen deficit) can have different underlying causes, including impaired ventilation (hypoxemia), inadequate oxygen-carrying capacity of the blood (anemia), reduced tissue blood flow (hypoperfusion due to impaired capillarization or vascular tone) or increased metabolic rate / mitochondrial oxygen consumption [32]. Future studies are warranted to elucidate the effects of altered oxygen levels in the tissue microenvironment on cardiometabolic health, as well as the underlying mechanisms, taking the severity, duration and pattern of hypoxia exposure into account. Compared with studies investigating the effects of altered pO_2_ on AT and SM, there are very few studies that examined the impact of low oxygen availability on liver metabolism and the expression/secretion of hepatokines. A better understanding of the metabolic impact of altered tissue oxygenation can provide valuable insights into the complex interplay between environmental and biological factors involved in the pathophysiology of obesity-related cardiometabolic derangements and may contribute to the development of novel strategies targeting oxygen signaling to prevent and treat obesity-related cardiometabolic complications.

## Data Availability

No datasets were generated or analysed during the current study.

## Abbreviations

AMPK: AMP-activated protein kinase
ATP: Adenosine triphosphate
AT: Adipose tissue
FFA: Free fatty acid
GDF: Growth and differentiation factor
HFD: High-fat diet
IL: interleukin
pO_2_: Oxygen partial pressure
PPARγ: Peroxisome proliferator-activated receptor γ
SM: Skeletal muscle
SPARC: Secreted protein acidic and rich in cysteine
T2DM: Type 2 diabetes mellitus
TNF-α: Tumor necrosis factor α
VEGF: Vascular endothelial growth factor

## Code availability

N/A.
